# The Emerging Workforce of International University Student Workers: Injury Experience in an Australian University

**DOI:** 10.3390/ijerph15030456

**Published:** 2018-03-06

**Authors:** Yahya Thamrin, Dino Pisaniello, Cally Guerin, Paul Rothmore

**Affiliations:** 1 Department of Occupational Safety and Health, Faculty of Public Health, Hasanuddin University, Makassar 90245, Indonesia; yahyathamrin@yahoo.com; 2 School of Public Health, Faculty of Health and Medical Sciences, University of Adelaide, Adelaide 5005, Australia; paul.rothmore@adelaide.edu.au; 3 Faculty of Arts, University of Adelaide, Adelaide 5005, Australia; cally.guerin@adelaide.edu.au

**Keywords:** international students, university, worker, injury, risk factor

## Abstract

International university students are a growing section of the workforce and are thought to be at greater risk of injury. Qualitative studies have highlighted vulnerabilities, but there is a shortage of quantitative research exploring the injury experience and associated risk factors of this emerging issue. In this study, a total of 466 university student workers across a range of study programs in a single Australian university completed an online survey, with questions relating to their background, working experience, training and injury experience. Risk factors for injury were explored in a multivariate statistical model. More than half had not received any safety training before they started work, and 10% reported having had a work injury. About half of these injuries occurred after training. Statistically significant risk factors for injury included working more than 20 h per week (adjusted odds ratio 2.20 (95% CI 1.03–4.71) and lack of confidence in discussing safety issues (AOR 2.17; 95% CI 1.13–4.16). The findings suggest the need for a more engaging and effective approach to safety education and a limit on working hours. This situation is a moral challenge for universities, in that they are effectively sponsoring young workers in the community. It is recommended that longitudinal studies of international student workers be conducted.

## 1. Introduction

International (foreign) students represent a significant and rising proportion of young workers in many countries. In 2016, there were over 300,000 international students enrolled in the higher education sector in Australia, a 13% increase over the previous year [1]. At the same time, international students may be at greater risk for occupational injury than their domestic peers [2]. Their vulnerability may be due to cultural and language barriers, fewer work choices, relatively poorer working conditions and, in many cases, financial pressures [3,4,5]. As such, they pose an emerging challenge for occupational health and safety (OHS) regulators.

A number of studies have explored students’ and international students’ vulnerability in the workplace [4,6,7]. These have been essentially qualitative. Nyland and coworkers [4] have highlighted exploitation (not attributed to racism), limited understanding by students of rights and support services, the need for education suppliers to provide relevant information and appropriate regulation. Anderson and coworkers [3] concluded that international student working experiences were similar to those of migrant workers, including their experience of illegal work. Ou and Thygerson [8] examined occupational injuries among university student employees using a quantitative approach but did not differentiate international students. This study found that heavy workload, work stress, and physical hazards exposure were the main contributing factors to injuries among university student workers. 

The profile of young workers’ injury experience may be gleaned from worker compensation statistics [9]. However, there is no specific occupational code for international students, and under-reporting for young workers, particularly females, is also a potential issue. Population survey methods are an alternative and more sensitive indicator of injury experience [10].

There is scarcity of quantitative research on international students’ working experience and training experience, together with the risk factors of injury among this sub-group of young workers.

Using a quantitative online survey approach, the aims of this study were to describe jobs, safety training, injuries and associated risk factors for international university student workers. This information will assist universities in raising awareness of student worker safety issues and in the development of appropriate interventions. 

## 2. Methods 

Rather than survey student workers in their workplaces, students were sampled in a familiar and common environment—namely a University portal. By preserving anonymity, we considered that students would be more likely to share information about themselves, their experiences and perceptions about safety at work.

### 2.1. Study Design and Data Collection

An online questionnaire, in a cross-sectional study design, was used for data collection. The survey of international students was conducted at the University of Adelaide using SurveyMonkey software (SurveyMonkey Inc., Palo Alto, CA, USA) in the second half of 2014. As there was no previously published survey instrument for international students, the questionnaire was designed in collaboration with the International Students Centre (ISC) of the University. After a pilot with a number of international students (*n* = 29, across a range of faculties) for intelligibility and logical flow, a survey invitation with its online link was distributed by the ISC to all international students at the University. The survey invitations were sent in two waves. In the first wave, the invitations were sent to the international students’ email address with the subject heading “Invitation for survey for international students”. This email included the survey information sheet, a consent form and the survey questionnaire. A follow-up, reminder email, was sent three weeks later. 

The survey was approved by the University of Adelaide Human Research Ethics Committee (Approval number HS-2013-045). The questionnaire is available as Supplementary Materials.

### 2.2. Participants

Information about the survey indicated that it was only for enrolled international students who had experience of working while studying in Australia. Both undergraduate and postgraduate students were eligible. 

### 2.3. Statistical Methods

Microsoft Excel Software (Microsoft Corporation, Redmond, WA, USA) was used for descriptive statistics. The IBM Statistical Package for the Social Sciences (SPSS) Version 21 (IBM Corporation, Armonk, NY, USA) was used for analytical statistics. The principal outcome variable in the study was student injury experience (“Have you ever had an injury at work while studying in Australia?”). Twenty independent variables, categorised into demographic factors, academic factors, working experiences and safety training experiences were assessed in terms of their association with the outcome variable of injury experience. 

Bivariate analysis using chi-square test (a two-tailed *p*-value of 0.05) and odds ratios (OR) were performed to determine the association between variables. Independent variables that were statistically significant and associated with the outcome variable in the bivariate analysis were included in a multivariate logistic regression model to identify important predictor factors. 

## 3. Results

The University of Adelaide is a medium-sized university with approximately 26% international students (approximately 5500 in 2015) [11]. 

Seven hundred and nineteen students took part in the survey, generating 466 complete responses. Only complete responses were used for data analysis. The majority of respondents came from China, Malaysia, Indonesia and Vietnam. 

### 3.1. Descriptive Analysis

Table 1 presents information on international student participant demographics, enrolment and financial factors, working experiences and safety training experiences and injury experiences.

#### 3.1.1. Working Experiences 

More than 50% of respondents were engaged in part-time work and approximately 39% of the participants had experience in casual jobs. Only 5.4% of this group had been involved in seasonal employment. 

More than 42% of participants reported that they had working experiences in the restaurant sector. There were 21.1% in the supermarket/grocery/shop sector, followed by the cleaning and agriculture sectors, with percentages at 12.6% and 5.8%, respectively. Approximately 18% of respondents revealed that they worked in another category, including education, human services, medical, nursing home, tutorial, education, university, hotel, marketing, accounting, sales and dental clinics. 

Turning to working hours, the vast majority of students worked less than 20 h per week; only 12.9% of them devoted 20 h or more per week to the workplace.

#### 3.1.2. Safety Training Experiences

With respect to training, 61% had not received any safety training before they started work. Amongst the remaining 39% who had received training from one or more sources, the university was the main provider (50%), followed by the workplace (41%), high school (9%) and technical college (TAFE) (10%). Of the 179 who had received training, 61 (34%) had no assessment. Regarding the format of safety training, 72% reported face-to-face safety training, 31% paper-based, 28% computer-based and 19% video-based training. 

#### 3.1.3. Injury Experiences

It can be seen from Table 1 that 48 students (10.3%) had experienced an injury in the workplace. More than 43% of these individuals experienced the injury after completing safety training. 

Half of the students reporting a work injury had experienced only one injury. About 21% of students recorded that they had been injured twice, and two percent had experienced work injuries on three occasions. Cuts and lacerations were the most common injury type (70%), followed by burns (38%), strains (17%) and injuries from slips/falls (17%).

### 3.2. Factors Associated with Injury Experiences

Table 2 provides information regarding the relationship between twenty independent variables and reported injury experience as the outcome variable. This table indicates there was a positive association between injury experience and being in the second year of study, with an unadjusted odds ratio (OR) of 3.06, 95% confidence interval (CI) 1.27–7.35.

Other significant associations with injury were: a perception of unfair wages, (OR of 2.71, 95% CI 1.45–5.06); working 20 h or more per week, (OR 2.57, 95% CI 1.25–5.28); and lack of confidence in discussing safety issues, (OR 2.61, 95% CI 1.40–4.88). 

Potential injury predictor variables found to be significant in the bivariate analysis were included in a logistic regression model generating adjusted odds ratios (AOR). It can be seen from Table 2 that there remained a significant association between international students’ injury experiences and being in the second year of study (AOR 2.69, 95% CI 1.04–6.96); having a perception of unfair wages (AOR 2.42, 95% CI 1.24–4.71); working 20 h or more per week (AOR 2.20, 95% CI 1.03–4.71); and lack of confidence in discussing safety issues (AOR 2.17, 95% CI 1.13–4.16).

## 4. Discussion

This is the first study to examine injury experience and its predictive factors among an increasing and vulnerable workforce of international student workers using a quantitative approach.

The discrepancy in the number of participating students and the number of complete responses occurred for two reasons. Firstly, the online survey allowed participants to skip questions for which they were unwilling to share information Secondly, although the online information indicated that the survey was only for those with working experiences, international students could participate in the online survey whether they had this experience or not. Participants who lacked this experience could not complete all the questions. Thus, only those respondents with complete responses (466) were included in the analysis. It is important to mention that there were some questions that allowed respondents to choose more than one option. Hence, in some cases total responses exceed 466.

### 4.1. International Students’ Working Conditions

This study found that most international students worked in a part-time job (55.3%), as distinct from casual (39.2%) and seasonal (5.4%). Most of the respondents worked in the restaurant supermarket, and cleaning industries; more than 90% of them worked indoors.

The findings are consistent with the qualitative studies of Nyland and coworkers [4] and Anderson and coworkers [6], both of whom maintained that a large number of international students work in the restaurant sector and other sectors that are commonly undesired by local employees. More broadly, the literature suggests that such workers frequently undertake low-status jobs, and work in substandard conditions. In addition, they may be susceptible to exploitation, since in many cases these workers are facing financial problems and lack a comprehensive understanding of safety arrangements in Australia [12]. 

However, this study found that a small percentage of international students (13%) worked 20 h or more per week, which is stated as the maximum work commitment outside of study allowable under their visa conditions. In a recent survey of temporary migrant workers in Australia, Berg and Farbenblum found working hours for international students to be similar to those observed in this study [13]. This finding is not consistent with Anderson and coworkers [14], which indicated that many international student workers in the UK breach their visa conditions or “bend the rules” for working over 20 h a week in illegal employment sectors. The reason for the different results between the UK study and the Australian study is not clear and needs further research.

### 4.2. Lack of Safety Training Experiences

Turning to safety training experience, the findings of this study provide important insights into the effectiveness of international students’ safety training experiences. Although provision of safety induction training is a legal requirement; more than 60% of the participants claimed that they did not receive any safety training before commencing work.

Unfortunately, more than 40% of students with training experienced injury after having safety training, indicating that the training may well have been inadequate. Relatedly, the results from bivariate analysis (Table 2) indicate that safety training experience was not significantly associated with injury. This is consistent with Zierold and Anderson [15], who claimed that safety training was not significantly associated with severe injuries among young workers. 

These findings, and the observation that only 58% of international students were confident in discussing safety matters, beg the question of the effectiveness of both university and workplace safety training for international students. 

It is widely assumed that educational programs have the potential to foster safety knowledge among students [16,17]. However, from the evidence in this study, it cannot be assumed that simply providing safety knowledge will automatically lead to the adoption of safe behaviour and a subsequent reduction of injuries in the workplace [18]. In line with Blair and coworkers [19], the current study strengthens calls for safety education and training to be more effective in influencing safety attitudes and behaviours. Additionally, this research suggests that more can be done to improve university–industry awareness with respect to safety training [18]. 

### 4.3. Injury Experience and Important Predictive Factors

Cuts and burns were the most common injuries. This is to be expected, since most of participants worked in the restaurant sector and the retail trade. Brosnan and Loudoun [20] reported that more than half of the teenage Australian labour force were employed in retail, accommodation, cafés and restaurants, where cuts and burns are often the result of processing, packing, cooking and serving. 

Four independent variables were found to be statistically significantly associated with students’ injury experiences: namely being in the second year of study (AOR 2.69); perceived unfair wages (AOR 2.42); working 20 h or more (AOR 2.20); and lacking confidence in discussing safety issues (AOR 2.17). 

With regard to year of study, there appear to be no comparable findings in the literature specific to international students. However, according to Gohn and coworkers [21], the main factors that may influence attrition of second year students include adjustment to stress, grade satisfaction, time management and financial management. Thus, it is plausible that students in their second year of study may be at greater risk than other students. However, it is more likely that the accident/injury occurred in the first year of study but was reported in the second year when the survey was conducted. The phenomenon of injuries occurring more frequently in unfamiliar environments is well recognized. Perception of wage unfairness (43%) was a significant predictive factor. While not explicit in our survey, it is likely that perceptions of wage unfairness and working hours may be associated with low levels of job satisfaction. Previous studies have reported that those who were dissatisfied with their work were almost three times more likely to report pain and four times more likely to experience an injury [22,23].

Focusing on weekly working hours, the findings are consistent with the US research about occupational injuries among university students, specifically, that a heavy workload is a risk factor for student employees experiencing one or more occupational injuries [8]. 

Our previous research with incoming university students in South Australia showed a significant association between safety training and skill and confidence in discussing safety issues [2]. Looking solely at international students, it is now revealed that students who are less confident about discussing safety issues are twice as likely to have experienced a workplace injury (AOR 2.17). 

One limitation of this study is that it was conducted in only one university, and at one point in time. However, the characteristics of international students at the University of Adelaide are similar to international students elsewhere in Australia [24]. Furthermore, it was not possible to determine the actual proportion of international students at the University of Adelaide with work experience in Australia, and thus eligible for inclusion. We were also unable to determine the severity of injury and consequences of the injury. Finally, we were unable to establish a causal relationship due to the cross-sectional nature of our study.

## 5. Conclusions

Firstly, it can be concluded that many, if not most, international students do not receive any safety training before commencing work. Secondly, for those who do have training, a significant proportion do go on to have an injury. Thirdly, it is evident that many international students lack confidence in discussing safety matters, which may include reporting of hazards, injuries or symptoms. These three findings suggest the need for a more engaging, effective and perhaps culturally sensitive approach to safety education for international student workers. This is also a moral challenge for universities, in that they are effectively sponsoring young workers in the community, and have a duty of care beyond discipline-specific education.

Finally, working more than 20 h per week appears to double the risk of workplace injury. There is a need to raise awareness of the safety issues associated with non-compliance with visa conditions on working hours.

It is recommended that longitudinal studies of international student workers be conducted, and the impacts of workplace injuries be investigated, in order to prevent injuries among this increasing large, and emerging, section of the workforce. 

## Figures and Tables

**Table 1:** International student worker characteristics and experiences.

Characteristic Data	Number of Students	% of Total Respondents (*n* = 466)
**Student demographics**		
Mean age 24.9 years, median age 24 years		
Females	246	52.8
Married	85	18.2
Have children	53	11.4
Have family members with them	100	21.5
**Academic factors**		
Faculty:		
Engineering, Computer and Mathematics	124	26.6
Health Sciences	44	9.40
Humanities and Social Sciences	39	8.40
Sciences	75	16.1
The Professions	184	39.5
Study program:		
Undergraduate	171	36.7
Study Abroad or Exchange program	50	10.7
Master	190	40.8
PhD	55	11.8
Years into study program:		
1st year	151	32.4
2nd year	157	33.7
3rd year	85	18.2
4th year	73	15.7
Financial support:		
Private	323	69.3
Partial scholarship	31	6.70
Full scholarship	112	24.0
**Working experiences**		
More than one job	56	12.0
Sector of industry:		
Restaurant	247	42.1
Supermarket/grocery/shop	124	21.1
Cleaning	74	12.6
Agriculture	34	5.8
Perception of wages (not fair)	199	42.7
Working more than 20 h/week	60	12.9
Outdoor workers	36	7.7
Not working under supervision	142	30.5
**Safety training experiences**		
No safety training	286	61.4
No assessment for training	61	34.1
**Injury experiences**		
Any injury	48	10.3
After safety training	21	43.8
Before safety training	27	56.3
**Other**		
Lacking confidence in discussing safety	197	42.3

**Table 2:** Predictors of injury experience among international student workers.

Independent Variables	Injury Experience			
	Unadjusted		Adjusted	
	OR	95% CI	OR	95% CI
**Student demographics**				
Age (>24 years), median split	1.16	0.63–2.13		
Gender (being female)	0.97	0.53–1.76		
Marital status (married)	0.88	0.39–1.97		
Have children	1.13	0.46–2.79		
Have family members with them	0.83	0.39–1.77		
**Academic factors**				
Faculty:				
Engineering, Computer and Mathematics	1.00			
Health sciences	2.27	0.94–5.49		
Humanities and Social Sciences	2.85	0.65–12.61		
Sciences	0.75	0.28–1.98		
The Professions	0.79	0.36–1.72		
Year of study:				
1st year	1.00			
2nd year	3.06 *	1.27–7.35 *	2.69 *	1.04–6.96 *
3rd year	1.78	0.82–3.90		
4th year	2.09	0.81–5.36		
Study program:				
Undergraduate	1.00			
Study Abroad or Exchange program	0.91	0.32–2.58		
Master	1.57	0.36–6.92		
PhD	0.73	0.26–2.01		
Financial support:				
Private	1.00			
Partial scholarship	1.57	0.74–3.36		
Full scholarship	0.72	0.22–1.84		
**Working experiences**				
Number of jobs (more than 1)	1.28	0.55–3.02		
Sector of industry:				
Restaurant	1.00			
Supermarket/grocery/shop	0.51	0.12–2.26		
Cleaning	0.64	0.14–3.04		
Agriculture	0.52	0.10–2.58		
Perception of wages (not fair)	2.71 *	1.45–5.06 *	2.42 *	1.24–4.71 *
Working hours/week (20 or more)	2.57 *	1.25–5.28 *	2.20 *	1.03–4.71 *
Working condition (outdoor)	0.91	0.31–2.70		
Not working under supervision	0.93	0.48–1.80		
**Safety training experiences**				
No safety training	1.44	0.76–2.72		
No assessment for training	2.42	0.83–7.01		
Lacking confidence in discussing safety	2.61 *	1.40–4.88 *	2.17 *	1.13–4.16 *

* Statistically significant, *p* < 0.05; Statistically significant for bivariate analysis; Statistically significant for multivariate analysis.

## Supplementary Materials

The following are available online at http://www.mdpi.com/1660-4601/15/3/456/s1, Survey questionnaire: International Students as Young Migrant Workers in South Australia: The Role of the University in OHS Awareness and Education.
