# Supplementary material for: The Emerging Workforce of International University Student Workers: Injury Experience in an Australian University
Source: Int J Environ Res Public Health. 2018 Mar 6;15(3):456. doi: 10.3390/ijerph15030456 (PMC5877001; doi:10.3390/ijerph15030456)
Supplement: Supplementary file 1 [file ijerph-15-00456-s001.pdf]

International Journal of
Environmental Research and Public Health

MDPI

__Online survey questionnaire__

Project title:

International Students as Young Migrant Workers in South Australia: The Role of the University in OHS Awareness and Education

A. DEMOGRAPHIC DATA

1. **Age :** ________ Years
2. **Gender :** Female ☐ Male ☐
3. **Study Program :** Undergraduate ☐ Master-Coursework ☐ Master - Research ☐ PhD ☐ Other (*please specify*) ________________
4. **Year of study :** 1st ☐ 2nd ☐ 3rd ☐ 4th ☐ Other (*please specify*) ________________
5. **Financial Support :** Private ☐ Partial Scholarship ☐ Full Scholarship ☐
6. **Which country are you from?** ________________
7. **In which faculty in the university do you study?**
   Humanities and Social Sciences ☐ The Professions ☐ Sciences ☐
   Engineering, Computer and Mathematics ☐ Health Sciences ☐
8. **Does your course involve a work placement or internship?** Yes ☐ No ☐
9. **Does your course involve:** **(*you can tick more than one*)** Laboratory work ☐ Field Trip ☐ Machinery workshop ☐
   Other workshop or practical tasks (*please specify*) ________________

B. STUDENT WORKING EXPERIENCE

(Only complete this section if you are currently working, or have been working while studying at a University in Australia)

10. What is your job status? Casual ☐ Part-time ☐ Seasonal ☐ Other ________

11. How many jobs do you have? One ☐ More than one ☐
(if more than one please fill the data for the primary job)

12. What is the sector of industry? Cleaning ☐ Supermarket/Grocery/Shop ☐
Agriculture ☐ Restaurant ☐ Other ________

13. Are exposed to any hazards at work? Chemical ☐ Biological ☐ Repetitive movement ☐
Physical ☐ Psychosocial ☐ Other ________
(you can tick more than one)

14. The main reason for working: ***(you can tick more than one)***
To pay tuition fee ☐ To supplement living allowance ☐
To pay living cost ☐ To support family in home country ☐
To get extra money ☐ To improve my language ☐
To understand Australian culture ☐ Peer recognition ☐
Other ________

15. Do you think your wages are fair? Yes ☐ No ☐

16. On average, how many hours do you work per week? ________

17. How long have you been working there? ________

18. Working conditions: Mainly outdoor ☐ Mainly indoor ☐

19. Do you work under supervision? Yes ☐ No ☐

C. TRAINING EXPERIENCE

20. Have you ever had any training in occupational health and safety? Yes ☐ No ☐

21. If YES, did it occur at: University ☐ TAFE ☐ High School ☐

As part of paid or voluntary employment outside of school? ☐

22. What was the length of training? ____________ hours

23. Was it: Single brief session ☐ Formal course ☐

Ongoing/repeated training ☐

24. Format : Paper-based ☐ Computer-based ☐

Video-based ☐ Face-to-face presentation ☐

25. Were you assessed on the training? Yes ☐ No ☐

26. Do you think the training was Too much ☐ About right ☐ Too little ☐

D. INJURY EXPERIENCE

27. Have you ever had an injury at work? Yes ☐ No ☐

If NO, please go to the question No. 34

28. If YES, did you receive OHS training before or after the injury? Before ☐ After ☐

29. How many times have you experienced injury at work?
Once ☐ Twice ☐ Three times ☐ More than three times ☐

30. What sort of injury that you ever had? (*please mention the primary one*)

____

31. What did you do to treat the injury?
Went to hospital emergency ☐ Visited GP ☐

Did not do anything ☐ Other ______________

32. If you had treatment in GP/hospital, how did you pay for the treatment?

Out of pocket payment ☐ Covered by OSHC ☐

Work Cover Compensation ☐ Other ____________

33. Did you report the injury to your supervisor or company? Yes ☐ No ☐

34. Have you ever witnessed a workplace accident? Yes ☐ No ☐

If NO, please go to the question No. 37

35. Did the accident result in a serious injury? Yes ☐ No ☐

36. Have you ever experienced any of following conditions in the workplace?

Discrimination Yes ☐ No ☐

Intimidation Yes ☐ No ☐

Exploitation Yes ☐ No ☐

Other (*please specify*) ____________

E. ACADEMIC INTERFERENCES

37. Do you feel that your work interferes with your study?

Never ☐ Sometimes ☐ Often ☐

If Never, please go to the question No. 40.

38. If SOMETIMES/OFTEN, how did it interfere?

Tiredness ☐ Time table clash ☐

☐ Not focused on study

39.Have you ever experienced any of following conditions?

Failure to submit assignment/task on time Yes ☐ No ☐

Stress Yes ☐ No ☐

F.STUDENT'S OHS PERCEPTIONS AND CONFIDENCE

40. Do you think you have the skills and confidence to discuss health and safety issues with your

lecturer, or other relevant person?

Yes ☐ No ☐

41. Do you feel strongly about any OHS issue? Yes ☐ No ☐

42. If YES, what issue? Personal security ☐ Bullying ☐

Workplace safety ☐ Other ________________ *(Please specify)*

G. ROLE OF UNIVERSITY IN RAISING OHS AWARENESS AND EDUCATION

43. Do you think the university has a responsibility to provide OHS education?

Yes ☐ No ☐

44. Where should OHS information be included?

Existing induction program for international students eg, EAP, IBP etc ☐

Separate workshop/additional OHS workshop ☐

As a part of normal curriculum at school ☐

Other ____________________

45. Do you think that health and safety has been included in your university study?

Yes ☐ No ☐

If NO, please go to the question No. 48

46. If YES, what kind of health and safety information provided? Rights and responsibilities ☐ Hazards ☐

Specific control measure ☐ Other ____________

47. Do you think it is directly relevant to your current job?

Yes ☐ No ☐

48. Do you think university needs to do more? Yes ☐ No ☐

49. Have you done the university OHS online induction course?

Yes ☐ No ☐

© 2018 by the authors. Submitted for possible open access publication under the terms and conditions of the Creative Commons Attribution (CC BY) license (http://creativecommons.org/licenses/by/4.0/).
